# Semi-Continuous Subcritical Water Extraction of Flavonoids from *Citrus unshiu* Peel: Their Antioxidant and Enzyme Inhibitory Activities

**DOI:** 10.3390/antiox9050360

**Published:** 2020-04-25

**Authors:** Dong-Shin Kim, Sang-Bin Lim

**Affiliations:** Department of Food Bioengineering, Jeju National University, Jeju 63243, Korea; feel567@naver.com

**Keywords:** *Citrus unshiu* peel, bioflavonoids, subcritical water extraction, hydrothermal hydrolysis products, antioxidant activity, enzyme inhibition activity

## Abstract

We extracted and hydrolyzed bioactive flavonoids from *C. unshiu* peel using subcritical water (SW) in a semi-continuous mode. The individual flavonoid yields, antioxidant and enzyme inhibitory activities of the SW extracts were analyzed. The extraction yields of hesperidin and narirutin increased with increasing temperature from 145 °C to 165 °C. Hydrothermal hydrolysis products (HHP), such as monoglucosides (hesperetin-7-*O*-glucoside and prunin) and aglycones (hesperetin and naringenin) were obtained in the SW extracts at temperatures above 160 °C. The sum of hesperidin and its HHP in the SW extracts was strongly correlated with antioxidant activities, whereas the contents of hesperetin and naringenin were strongly correlated with enzyme inhibitory activities. Hesperetin exhibited the highest antioxidant activities (2,2-diphenyl-1-picrylhydrazyl radical scavenging activity, ferric-reducing antioxidant power, and oxygen radical absorbance capacity), whereas hesperetin-7-*O*-glucoside exhibited the highest enzyme inhibitory activities (angiotensin-I converting enzyme (ACE) and pancreatic lipase (PL)). Naringenin exhibited the highest enzyme inhibitory activities (xanthine oxidase and α-glucosidase). PMFs (sinensetin, nobiletin, and tangeretin) also exhibited relatively high inhibitory activities against ACE and PL. This study confirms the potential of SW for extracting and hydrolyzing bioactive flavonoids from *C. unshiu* peel using an environmentally friendly solvent (water) and a shorter extraction time.

## 1. Introduction

*Citrus unshiu* is a major fruit grown in Jeju, Korea, and is widely used in the food industry for juice production. As the yield of citrus juice from fresh fruit is approximately 50% of the fruit by weight, large amounts of byproducts, such as peel, pulp, and seeds, are produced [1]. Citrus peel represents the major byproduct from the citrus juice industry. Citrus peel has been used to treat the common cold, indigestion, bronchial discomfort, etc., in Korean traditional medicine [2,3]. Citrus peel is a major source of citrus flavonoids, such as hesperidin, narirutin, and polymethoxyflavones (PMFs; e.g., sinensetin, nobiletin, and tangeretin), which have various functional properties, including antioxidant, antimicrobial, anticancer, anti-inflammatory, antiobesity, and antihyperglycemic activities [4,5,6].

**Figure 1:**
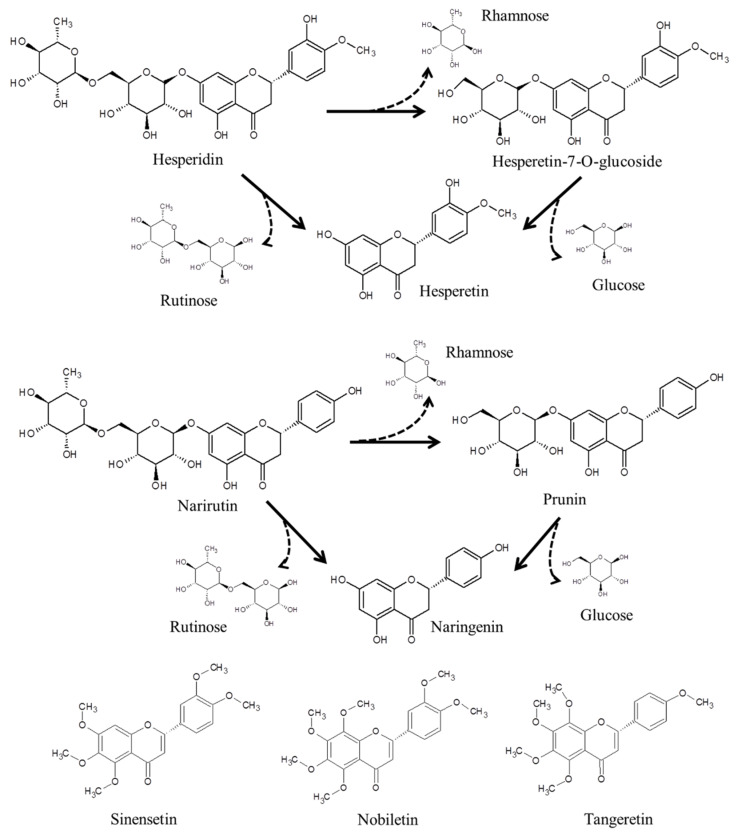
Chemical structures and hydrolysis pathways of citrus flavonoids.

Ethanol or methanol are frequently used in extraction of citrus flavonoids, but their use is limited by the toxicity of the solvent residue, strict legislation, and long extraction times [7]. Recently, high pressure and ultrasound-assisted extraction methods have been used to increase the extraction efficiency of citrus flavonoids, but it is still not possible to avoid the use of organic solvents [8]. As an alternative to organic solvent extraction, subcritical water extraction (SWE), an environmentally friendly technique, has been used to extract medium- or non-polar bioactive compounds from plant materials. SW is water that maintains a liquid state at a temperature of 100–374 °C under pressure. In the subcritical region, the dielectric constant associated with the polarity of water is reduced due to the breakdown of hydrogen bonds between the water molecules, which allows for the extraction of medium- or non-polar compounds, such as flavonoids [9]. SW also improves extraction efficiency by reducing viscosity and surface tension and increasing the self-diffusion of water [10]. Additionally, the subcritical condition increases the levels of ionic products of water, thus forming an acidic medium for hydrolysis reactions [11], and flavonoid diglucosides can be hydrolyzed to flavonoid monoglucosides and aglycones using SW (Figure 1).

There have been several studies on the extraction of phenolic compounds from *C. unshiu* using SW over the past decade. Cheigh et al. [11] studied the SWE of hesperidin and narirutin from citrus fruits by varying extraction temperature (110–200 °C) and static time (5–20 min) in batch mode. Kanmaz and Saral [12] extracted mandarin peel using SW and determined the effects of extraction temperature (50–180 °C) and static time (5 and 15 min) on total phenolic and flavonoid contents and the antioxidant activities of the SW extracts in batch mode. Lachos-Perez et al. [7] studied the effects of extraction temperature (110–150 °C) and flow rate (10–30 mL/min) on flavonoid yields from defatted orange peel using a semi-continuous SWE unit. Most studies reported to date conducted only single-factor experiments on extraction temperature and static time mainly using a batch-type extractor. In addition, various functionalities of the SW extracts and hydrolysates from *C. unshiu* peel have not been measured, and there have been no studies regarding which chemical compounds determine the functional properties of the SW extracts and hydrolysates.

In this study, the extraction parameters (extraction temperature and flow rate) were optimized using response surface methodology (RSM) to maximize the yields of five flavonoids, i.e., hesperidin, narirutin, sinensetin, nobiletin, and tangeretin, from *C. unshiu* peel using SW in semi-continuous mode. Functional properties, such as the antioxidant activities (2,2-diphenyl-1-picrylhydrazyl [DPPH] radical scavenging activity, ferric-reducing antioxidant power [FRAP], and oxygen radical absorbance capacity [ORAC]) and enzyme inhibitory activities (against xanthine oxidase [XO], angiotensin-I converting enzyme [ACE], α-glucosidase, and pancreatic lipase [PL]) of the SW extracts and individual citrus flavonoids, including their hydrolysis products, were also analyzed.

## 2. Materials and Methods 

### 2.1. Sample Preparation

*C. unshiu* Markovich fruits were purchased from a local citrus farm in Jeju, Korea. The peel was separated from the fruit, dried for three days at room temperature in the shade, ground into powder (20–50 mesh), and stored at 20 °C.

### 2.2. Chemicals

Hesperidin, hesperetin, narirutin, naringenin, DPPH, 2,4,6-tris(2-pyridyl)-*s*-triazine (TPTZ), ferrous sulfate heptahydrate, fluorescein sodium salt, N-hippuryl-his-leu hydrate (HHL), lung acetone powder from rabbit, xanthine oxidase from bovine milk, allopurinol, *ρ*-nitrophenyl-β-D-glucopyranoside (*ρ*-NPG), acarbose, α-glucosidase from Saccharomyces cerevisiae, *ρ*-nitrophenyl butyrate (*ρ*-NPB), lipase from porcine pancreas, orlistat, and 3-(*N*-Morpholino)propane sulfonic acid (MOPS) were purchased from Sigma Chemical Co. (St. Louis, MO). 2,2′-Azobis(2-methylpropionamidine) dihydrochloride (AAPH) was purchased from Acros organics (Geel, Belgium) and captopril was purchased from Tokyo Chemical Industry Co., Ltd. (Kita-ku, Tokyo, Japan). Sinensetin, nobiletin, and tangeretin were purchased from Avention (Yeonsu-gu, Incheon, Korea). Hesperetin-7-*O*-glucoside and prunin were purchased from Extrasynthese (Genay, France). Acetic acid was purchased from Junsei Chemical Co., Ltd (Chuo-ku, Tokyo, Japan) and HPLC grade acetonitrile and methyl alcohol were purchased from Daejung Chemicals & Metals Co., Ltd (Shiheung, Gyeonggi, Korea).

### 2.3. Subcritical Water Extraction (SWE)

Semi-continuous SWE was carried out using our laboratory-built apparatus (Figure S1). Samples consisting of 1 g of peel powder were mixed with sea sand (14–20 mesh; Wako, Chuoku, Tokyo, Japan) and loaded into a stainless-steel extractor (7.8 mm × 300 mm). Glass wool and filters (stainless steel, 5 μm pore size) were placed at both ends of the extractor. The extractor was connected to the line leading to a gas chromatography oven (Agilent Technologies, Santa Clara, CA) and heated to the desired temperature (145–175 °C). A high-performance liquid chromatography (HPLC) pump (Thermo separation products, Waltham, MA) was used for the passage of deionized and degassed water at a constant flow rate (0.75–2.25 mL/min) controlled by a metering valve (Parker Autoclave Engineers, Erie, PA). The pressure was maintained at 5 MPa using a pressure regulator (Tescom Corp. Elk River, MN). Extracts were collected for 15 min. Individual flavonoid concentrations were quantified using HPLC, and the functional properties were analyzed for each extract. The percentage soluble content (%) of each extract (as a solid) was calculated relative to the total solid content after drying at 105 °C.

### 2.4. Methanol Extraction

Samples consisting of 1 g of dried peel powder were extracted using 30 mL of pure methanol for 30 min at room temperature with stirring. The supernatant was recovered after centrifugation (10,000 rpm, 10 min), and the residue was re-extracted four more times. The extract was concentrated and filtered (0.45 μm) before a HPLC analysis.

### 2.5. Acid and Base Hydrolysis

Acid and base hydrolysis of the peel powders was performed as described previously [13] with some modifications. In the case of acid hydrolysis, samples consisting of 1 g of peel powder were hydrolyzed in 20 mL of 2 M HCl for 60 min at 85 °C with shaking (120 rpm). The hydrolysis solution was allowed to cool to room temperature, adjusted to pH 2.0 using 12 M NaOH, and then centrifuged at 10,000 rpm for 10 min. The supernatant was recovered, and the residue was washed two more times by adding 20 mL of neat methanol and vortexing for 1 min. The hydrolysate was then concentrated and filtered (0.45 μm) before the HPLC analysis.

For base hydrolysis, samples consisting of 1 g of peel powder were hydrolyzed in 20 mL of 2 M NaOH (containing 10 mM EDTA and 1% ascorbic acid) for 4 h at 25 °C with shaking (120 rpm). The hydrolysis solution was adjusted to pH 2.0 using 12 M HCl and then centrifuged at 10,000 rpm for 10 min. The supernatant was recovered and the residue was washed two more times with 20 mL of neat methanol with vortexing. The hydrolysate was then concentrated and filtered (0.45 μm) before the HPLC analysis.

### 2.6. HPLC Analysis

Hesperidin and its hydrothermal hydrolysis products (HHP) (hesperetin-7-*O*-glucoside and hesperetin), narirutin and its HHP (prunin and naringenin), and PMFs (sinensetin, nobiletin, and tangeretin) in the SW extracts were quantified using an HPLC system. The chromatographic peaks were separated using an Inertsil ODS-3V column (5 μm, 4.6 mm × 250 mm, GL Science, Tokyo, Japan). Aqueous 0.1% acetic acid solution (solvent A) and acetonitrile (solvent B) were used as the mobile phases. The injection volume was 10 μL, the flow rate was set to 1.0 mL/min, and the column temperature was 40 °C. Detection was performed at wavelengths of 290 and 330 nm. The gradient program of the mobile phase was as follows: 0 min 15% B, 8 min 25% B, 15 min 25% B, 35 min 65% B, 37 min 65% B, and 39 min 15% B. The chromatographic peaks of the flavonoids were confirmed by comparing the UV spectra and retention times with those of standard compounds [14]. Table S1 shows the regression equation for each standard compound analyzed.

### 2.7. Response Surface Design

The SWE of citrus flavonoids was optimized using RSM. A central composite design was selected to optimize the relationships between the response variables and two independent variables-temperature (X_1_: 145–175 °C) and flow rate (X_2_: 0.75–2.25 mL/min) (Table S2). The quadratic polynomial was used to predict the optimal extraction conditions based on experimental data with the following equation:

(1) $${Y=\beta }_{0}{+\beta }_{1}X_{1}{+\beta }_{2}X_{2}{+\beta }_{11}X_{1}{}^{2}{+\beta }_{22}X_{2}{}^{2}{+\beta }_{12}X_{1}X_{2}$$

where X_1_ and X_2_ are levels of independent variables, Y is the response variable, and *β*_0_, *β*_1_, *β*_2_, *β*_11_, *β*_22_, and *β*_12_ are coefficients. The fit of each model was evaluated via analysis of variance (ANOVA) using Design-Expert^®^ software (Stat-Ease Inc., Minneapolis, MN, USA).

### 2.8. Antioxidant Activity Measurement

The antioxidant activities—DPPH radical scavenging activity, FRAP, and ORAC—of the SW extracts and individual flavonoids were measured. DPPH radical scavenging activity was measured as described previously [15] with some modifications. Briefly, a 0.5-mL sample was mixed with 2.0 mL DPPH solution (0.2 mM), and the mixture was allowed to react for 30 min in the dark. The absorbance was then measured at 517 nm. DPPH radical scavenging activity was expressed as mg ascorbic acid equivalents (AAE)/g dry sample.

FRAP was measured as described previously [15] with some modifications. Briefly, the FRAP reagent was prepared by mixing 300 mM acetate buffer, 20 mM FeCl_3_·6H_2_O, and 10 mM TPTZ at a ratio of 1:1:10 (*v*/*v*). A 0.2-mL sample was mixed with 3 mL of FRAP reagent and 0.3 mL of distilled water and allowed to react for 30 min at 37 °C. The absorbance was then measured at 595 nm. FRAP was expressed as mmol ferrous sulfate equivalents (FSE)/100 g dry sample.

ORAC was measured as described previously [15] with some modifications. Briefly, a 0.025-mL sample was mixed with 0.15 mL of fluorescein sodium salt solution (78 nM) and allowed to react for 15 min at 37 °C. Then, 0.025 mL of AAPH (250 mM) was added to the solution, and the fluorescence was measured every 3 min for 120 min (excitation, 485 nm; emission, 535 nm). The area under the curve was calculated for each sample and compared to that of a Trolox standard. ORAC was expressed as mg Trolox equivalents (TE)/g dry sample.

### 2.9. Enzyme Inhibitory Activity Measurement

Enzyme inhibitory activities (against XO, ACE, α-glucosidase, and PL) in the SW extracts and individual flavonoids were measured. A stock solution of each flavonoid standard was prepared by dissolving the standard in dimethyl sulfoxide (DMSO), and the working solution was prepared by dissolving standards in suitable buffer solutions for each experiment. The final DMSO concentration was ≤ 5% (*v*/*v*), which did not affect enzyme activities.

XO inhibitory activity was measured as described previously [16] with some modifications. Briefly, a 0.1-mL sample was mixed with 0.3 mL of phosphate buffer (50 mM, pH 7.5) and 0.1 mL of 0.5 U/mL XO solution (in 50 mM phosphate buffer, pH 7.5). The reaction mixture was incubated for 15 min at 25 °C. Then, 0.2 mL of 2 mM xanthine solution was added, and the mixture was incubated for 30 min at 25 °C. The enzyme reaction was stopped with 0.3 mL of 1 M HCl. The reaction mixture was diluted with distilled water, and the absorbance was measured at 290 nm. Allopurinol was used as a positive control.

ACE inhibitory activity was measured as described previously [17] with some modifications. Briefly, a 0.05-mL sample was mixed with 0.1 mL HHL solution (12.5 mM) and incubated for 5 min at 37 °C. Then, 0.15 mL of ACE solution (in 50 mM phosphate buffer, pH 7.3) was added, and the mixture was re-incubated for 60 min at 37 °C. The enzyme reaction was stopped with 0.25 mL of 1 M HCl, and the hippuric acid in the reaction mixture was extracted by adding 1.5 mL of ethyl acetate. After centrifugation at 4000 rpm for 5 min, 1.0 mL of the supernatant was transferred to a clean tube and evaporated. Then, 1 mL of distilled water was added to dissolve the dry residue, and the absorbance was measured at 228 nm. Captopril was used as a positive control.

The α-glucosidase inhibitory activity was measured as described previously [18] with some modifications. Briefly, a 0.05-mL sample was mixed with 0.1 mL of phosphate buffer (100 mM, pH 6.8) and 0.05 mL of α-glucosidase solution (1 U/mL, in 100 mM phosphate buffer, pH 6.8). The enzyme mixture was incubated for 15 min at 37 °C. Then, 0.1 mL of *ρ*-NPG (3 mM) was added, and the mixture was re-incubated for 10 min at 37 °C. The enzyme reaction was stopped with 0.45 mL of Na_2_CO_3_ (0.1 M), and the absorbance was measured at 405 nm. Acarbose was used as a positive control.

PL inhibitory activity was measured as described previously [19] with some modifications. Briefly, a 5 mg/mL enzyme solution was prepared by dissolving porcine PL in MOPS buffer (10 mM MOPS and 1 mM EDTA, pH 6.8). A 0.025-mL sample was mixed with 0.9 mL Tris-HCl buffer (100 mM Tris-HCl and 5 mM CaCl_2_, pH 7.0) and 0.025 mL of enzyme solution (5 U/mL). The enzyme mixture was incubated for 15 min at 37 °C. Then, 0.05 mL of *ρ*-NPB (10 mM) was added, and the mixture and re-incubated at 37 °C for 30 min. The absorbance was measured at 405 nm. Orlistat was used as a positive control.

### 2.10. Statistical Analysis

Differences between treatments were analyzed using Duncan’s multiple range test (*p* < 0.05 denotes statistical significance). Pearson correlation coefficients (*r*) representing the relationships between flavonoid contents and functional properties were also calculated. All statistical analyses were performed using SPSS software (SPSS Inc., Chicago, IL, USA).

## 3. Result and Discussion

### 3.1. Composition of Flavonoids in C. unshiu Peel

The flavonoid composition in the methanol extract of *C. unshiu* peel is shown in Table S3. The major flavonoids were hesperidin (50,027 μg/g dry sample) and narirutin (9284 μg/g dry sample). Monoglucosides and aglycones other than hesperidin and narirutin were not detected. The minor flavonoids were PMFs (0.3%) such as sinensetin (18.9 μg/ g dry sample), nobiletin (103.8 μg/g dry sample), and tangeretin (55.5 μg/g dry sample). The sum of all flavonoid contents was 59,490 μg/g dry sample.

### 3.2. Optimization of SWE Process

Table 1 presents the yields of hesperidin and its HHP (hesperetin-7-*O*-glucoside and hesperetin), narirutin and its HHP (prunin and naringenin), and PMFs (sinensetin, nobiletin, and tangeretin) in the SW extracts of citrus peel at different temperatures and flow rates. Table S4 presents the results of ANOVA and regression analysis for the five flavonoids. All regression models produced high *R*^2^ values (>0.966), low *p*-values (<0.05), and nonsignificant lack-of-fit *p*-values (>0.05), indicating that the regression models provided good fits. Moreover, smaller differences (<0.2) between adjusted and predicted *R*^2^ values, low %C.V. values (≤5%), and high adequate precision (>16) also indicated that the regression models provided good fits [20].

The optimal extraction conditions for maximizing the yields of the five flavonoids were estimated using the following second-order polynomial equations:

(2) $$Y(\mathrm{hesperidin})=37,098+2470X_{1}+6609X_{2}-2411{X_{1}}^{2}-1016{X_{2}}^{2}-204X_{1}X_{2}$$

(3) $$Y(\mathrm{narirutin})=6821+4.4X_{1}+1277X_{2}-68.5{X_{1}}^{2}+3.8{X_{2}}^{2}-50.6X_{1}X_{2}$$

(4) $$Y(\mathrm{sinensetin})=16.19-0.62X_{1}+2.01X_{2}-0.49{X_{1}}^{2}-0.69{X_{2}}^{2}-0.55X_{1}X_{2}$$

(5) $$Y(\mathrm{nobiletin})=83.37+2.51X_{1}+8.71X_{2}-1.44{X_{1}}^{2}-2.55{X_{2}}^{2}-0.59X_{1}X_{2}$$

(6) $$Y(\mathrm{tangeretin})=41.52+1.76X_{1}+8.01X_{2}-1.36{X_{1}}^{2}+0.02{X_{2}}^{2}-1.10X_{1}X_{2}$$

where *Y* is the yield, *X*_1_ is temperature, and *X_2_* is flow rate.

**Figure 2:**
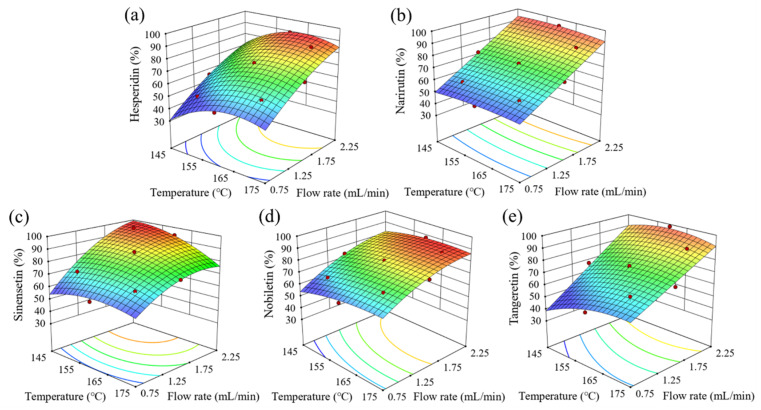
Response surface plots indicating the effects of extraction temperature and flow rate on flavonoid yields: (**a**) hesperidin, (**b**) narirutin, (**c**) sinensetin, (**d**) nobiletin, and (**e**) tangeretin.

Based on the models, the optimal extraction temperatures of hesperidin, narirutin, sinensetin, nobiletin, and tangeretin were 164.4 °C, 154.6 °C, 145.3 °C, 165.6 °C, and 160.5 °C, respectively, and the optimal flow rate was 2.25 mL/min (Table S4). The predicted yields of hesperidin, narirutin, sinensetin, nobiletin, and tangeretin at the optimal extraction conditions were 45,211, 8765, 18.7, 91.2, and 53.6 μg/g dry sample, respectively, which were 90.4%, 94.4%, 98.9%, 87.8%, and 96.6%, respectively, of the corresponding raw material contents. The optimal SWE conditions for simultaneous extraction of the five flavonoids were a temperature of 158.5 °C and a flow rate of 2.25 mL/min, which resulted in maximum yields of 88.7%, 94.3%, 94.2%, 87.1%, and 96.4% for hesperidin, narirutin, sinensetin, nobiletin, and tangeretin, respectively. The relationships between the responses (flavonoid yields) and experimental variables (extraction temperature and flow rate) are presented in the response surface plots in Figure 2.

### 3.3. Effects of Extraction Parameters on Individual Flavonoid Yields

Table 1 and Figure 2 present individual flavonoid yields in the SW extracts at different temperatures and flow rates. The hesperidin yield increased with increasing extraction temperature from 145 °C to 165 °C. This was because the dielectric constant decreases as the temperature increases, which reduces the polarity of water. The dielectric constant of water at 20 °C is 80.2, but it decreases to 44.9 and 40.9 as the temperature increases to 145 °C and 165 °C, respectively [21]. Céliz et al. [22] reported that the solubility of hesperidin increased by more than fivefold when the water temperature increased from 110 °C to 160 °C. Lachos-Perez et al. [7] also reported that the hesperidin yield from defatted orange peel increased by 1.8–4.1-fold when the extraction temperature increased from 110 °C to 150 °C at different flow rates. However, the hesperidin yield decreased at temperatures higher than 165 °C because hesperidin was hydrolyzed to its monoglucoside (hesperetin-7-*O*-glucoside) or aglycone (hesperetin) [23].

By contrast, the impact of temperature on the narirutin yield was not greater than that on the hesperidin yield due to the higher solubility of narirutin (compared to hesperidin) in water [24]. This was consistent with the low optimal extraction temperature (154.6 °C) of narirutin compared with that of hesperidin (164.4 °C) (Table S5). Park et al. [25] reported that narirutin is water-soluble, whereas hesperidin is hardly soluble in water. The HHP of narirutin, such as prunin and naringenin, were also detected at temperatures higher than 160 °C, and their yields increased at higher temperatures and lower flow rates.

For PMFs, the nobiletin yield increased with increasing temperature from 145 °C to 165 °C and decreased slightly at temperatures above 165 °C at a flow rate of 2.25 mL/min (Figure 2). The tangeretin yield also increased with increasing temperature from 145 °C to 160 °C at a flow rate of 2.25 mL/min. However, the sinensetin yield was highest at 145 °C due to its thermal instability [26]. The optimal extraction temperature of sinensetin was low (145.3 °C), whereas those of nobiletin (165.6 °C) and tangeretin (160.5 °C) were high. Zhang et al. [26] reported that the degradation ratio of sinensetin as represented by demethylation during hot-air drying was higher than those of nobiletin and tangeretin.

Table 1 and Figure 2 also show the effects of water flow rates on flavonoid yields. The yields of individual flavonoids increased significantly with an increasing flow rate from 0.75 to 2.25 mL/min under subcritical conditions. This was because higher flow rates increased the extraction yield by increasing the concentration gradient, superficial velocity, and mass transfer rate between the extraction solvent and the sample matrix during SWE [27,28]. In particular, the extraction yield of narirutin was greatly affected by the flow rate because narirutin is more soluble in water compared to the other flavonoids. On the other hand, the hesperidin yield was influenced by the flow rate as well as temperature because it is less soluble in water compared to narirutin [25]. The PMF yields were also significantly affected by flow rate because they are less soluble in water.

The extraction yield of total soluble solids increased with increasing temperature and flow rate as well (Table 1). The sum of individual flavonoid contents in the raw material was 59.49 mg/g dry sample (Table S3), but that in the SW extract at 160 °C and 2.25 mL/min increased to 90.1 mg/g dry extract, corresponding to 9.0% of the extract.

### 3.4. Comparison of Flavonoid Compositions in the SW Extract and Acid and Base Hydrolysis

The flavonoid composition in the SW extract at 175 °C and 1.5 mL/min was compared with those in the acid and base hydrolysates of citrus peel (Table 2). The yields of hesperidin and narirutin in the SW extract were 2.15–2.87-fold and 4.07–5.31-fold higher than those in the acid and base hydrolysates, respectively. Some of the hesperidin and narirutin were converted to their HHP in the SW extract, indicating that citrus flavonoid diglucosides can be hydrolyzed into highly functional low-molecular-weight compounds when they are treated with SW instead of an acid or base.

In the case of the acid hydrolysate, monoglucosides (hesperetin-7-*O*-glucoside and prunin) and aglycones (hesperetin and naringenin) were produced via the acid hydrolysis of glycosidic bonds between aglycone and sugar [29]. However, the yields were very low, indicating that the generated aglycones had been decomposed into smaller molecules. In the case of the base hydrolysate, HHP such as monoglucosides and aglycones were not detected at all. This was due to the hydrolysis of esters and ether bonds, but not glycosidic bonds, via base hydrolysis [29]. In the present study, several unknown peaks were detected in the base hydrolysate (Figure S2), which indicate that flavanones were converted to chalcone derivatives under basic conditions [30].

### 3.5. Antioxidant and Enzyme Inhibitory Activities in the SW Extracts

Table 3 presents the antioxidant activities (DPPH radical scavenging activity, FRAP, and ORAC) and enzyme inhibitory activities (against XO, ACE, α-glucosidase, and PL) in the SW extracts from *C. unshiu* peel processed at different temperatures and flow rates. DPPH radical scavenging activity in the SW extracts increased with increasing temperature and flow rate. The highest DPPH radical scavenging activity was obtained at 160 °C and 2.25 mL/min as well as 175 °C and 1.5 mL/min. FRAP and ORAC also exhibited similar trends as DPPH radical scavenging activity. Lachos-Perez et al. [7] reported that the antioxidant capacities (DPPH radical scavenging activity, FRAP, and ORAC) of the SW extracts from defatted orange peel increased with increasing temperature (110–150 °C). Kanmaz et al. [12] reported that the SW extracts from mandarin peel exhibited high antioxidant activities (FRAP and cupric-reducing antioxidant capacity) at higher extraction temperatures and longer extraction times.

The enzyme inhibitory activities, such as those against XO, ACE, α-glucosidase, and PL, were also measured (Table 3). These activities are related to anti-gout, anti-hypertension, anti-diabetes, and weight-control effects, respectively [16,17,18,19]. The enzyme inhibitory activities in the SW extracts against XO, ACE, and α-glucosidase increased with increasing temperature and flow rate, and the highest activity was obtained at 175 °C and 1.5 mL/min. Nile et al. [31] reported that XO and α-glucosidase inhibitory activities in *Withania somnifera* L extracts processed using SW increased with increasing temperature from 100 °C to 160 °C. In particular, the PL inhibitory activities in all SW extracts were higher compared to other enzyme inhibitory activities probably due to the high content of citrus flavonoids [32].

### 3.6. Correlations between Flavonoid Yields and Functional Properties of the SW Extracts

To determine which flavonoids affect the functional properties (antioxidant and enzyme inhibitory activities) of the SW extracts, Pearson correlation coefficients (*r*-values) representing the associations between flavonoid yields and functional properties were calculated (Table S6). The sum of individual flavonoids was strongly correlated with DPPH radical scavenging activity (*r* = 0.911), FRAP (*r* = 0.798), and ORAC (*r* = 0.841), indicating that the total flavonoid content in the SW extracts has a large effect on antioxidant activities. The content of hesperidin, the major flavonoid in the peel, was also more strongly correlated with antioxidant activities (*r* = 0.725–0.857) compared to narirutin (*r* = 0.479–0.685). The sum of HHP contents (hesperetin-7-*O*-glucoside, hesperetin, prunin, and naringenin) was more strongly correlated with inhibitory activities against XO (*r* = 0.826), ACE (*r* = 0.798), α-glucosidase (*r* = 0.830), and PL (*r* = 0.795) than with antioxidant activities (*r* ≤ 0.719), indicating that HHP formed at high temperatures have a marked effect on enzyme inhibitory activities. Individual HHP were also more strongly correlated with inhibitory activities against XO (*r* ≥ 0.736), ACE (*r* ≥ 0.666), α-glucosidase (*r* ≥ 0.708), and PL (*r* ≥ 0.695) than were their precursors, hesperidin (*r* ≤ 0.602) and narirutin (*r* ≤ 0.393).

### 3.7. Antioxidant Activities of Individual Citrus Flavonoids

Table 4 shows the antioxidant activities (DPPH, FRAP, and ORAC) of individual flavonoids. Hesperidin and its HHP exhibited 1.94–2.57-fold higher DPPH radical scavenging activity and 3.66–4.42-fold higher FRAP than did narirutin and its HHP, but there was no marked difference in ORAC. Mhiri et al. [33] reported that the 2,2′-azinobis-(3-ethylbenzothiazoline-6-sulfonic acid) (ABTS) radical scavenging activity of hesperidin was 5.5-fold higher than that of narirutin due to the presence of a catechol group in the B-ring of the hesperidin molecule.

The antioxidant activities of monoglucosides (hesperetin-7-*O*-glucoside and prunin) and aglycones (hesperetin and naringenin) were high compared with those of diglucosides (hesperidin and narirutin), and among them, hesperetin exhibited the highest antioxidant activity. Flavonoid diglucosides exhibit lower antioxidant activities compared with their aglycones because one or more sugars are combined with aromatic hydroxyl groups in their molecular structures [34].

PMFs did not exhibit DPPH radical scavenging activity and FRAP and exhibited a relatively low ORAC. Barreca et al. [35] reported that PMFs in tangelo exhibited negligible antioxidant activities (DPPH radical scavenging activity, ABTS radical scavenging activity, FRAP, and hydroxyl radical scavenging activity).

Table S7 shows the theoretical antioxidant activities in the SW extracts calculated based on the antioxidant activities of individual flavonoids. All measured antioxidant activities were higher than the theoretical antioxidant activities in the SW extracts, which may have been due to the synergistic effects between flavonoids or unknown compounds produced under subcritical conditions. Plaza and Turner [10] reported that new antioxidants were produced by Maillard, caramelization, and thermal oxidation reactions under subcritical conditions. Further research is needed on antioxidant synergism between citrus flavonoids, degradation products, and unknown compounds in the SW extracts.

### 3.8. Enzyme Inhibitory Activities of Individual Citrus Flavonoids

The enzyme inhibitory activities of individual flavonoids were also measured (Table 4 and Tables S8–S11). The inhibitory activities against XO and α-glucosidase exhibited by narirutin and its HHP were about twice those exhibited by hesperidin and its HHP due to the hydroxyl groups at the 4′-position of the B-ring in their molecules. The highest activities against both enzymes were exhibited by aglycones (hesperetin and naringenin), followed by monoglucosides (hesperetin-7-*O*-glucoside and prunin) and diglucosides (hesperidin and narirutin). In particular, naringenin exhibited a high inhibitory activity (IC_50_) of 129.3 mg/L against XO, which was not significantly different from the value of 116.7 mg/L for the positive control, allopurinol. The inhibitory activity (IC_50_) exhibited by naringenin against α-glucosidase was 72.2 mg/L, which was 18.9-fold higher than that (1366.9 mg/L) of the positive control, acarbose. Yuan et al. [36] reported that the inhibitory activities exhibited by flavonoids, such as kaempferol, quercetin, and naringenin, against XO decreased with increasing glycosylation. Hesperidin and its HHP exhibited relatively low inhibitory activities and PMFs exhibited almost no activities against XO and α-glucosidase due to the presence of methyl groups in their molecular structures. Lin et al. [37] also reported that the methylation of hydroxyl groups at the B-ring of some flavonoids (luteolin and kaempferol) greatly reduced XO inhibitory activity.

The inhibitory activities against ACE were higher for hesperidin and its HHP than for narirutin and its HHP, and those for monoglucosides and aglycones were 8.7–12.9-fold and 3.8–4.2-fold higher, respectively, than those for diglucosides. In particular, the ACE inhibitory activities exhibited by hesperetin-7-*O*-glucoside and prunin were the highest.

The inhibitory activity against PL was very high for hesperidin and its HHP, but very low for narirutin and its HHP. In particular, the inhibitory activities (IC_50_) exhibited by hesperetin-7-*O*-glucoside and hesperetin were the highest (84.4 and 104.6 mg/L, respectively). This was due to a specific structure (methylation in the 4′-position) found in hesperidin and its HHP, which directly inhibits PL. Buchholz and Melzig [38] also reported that the hydroxyl group at the 3′-position and the methyl group at the 4′-position of the B-ring in the hesperidin molecule favor inhibitory activity against PL. The SW extracts of citrus peel exhibited high inhibitory activities against PL probably due to the high content of hesperidin, which exhibits high inhibitory activity against PL (Table 3).

PMFs also exhibited relatively high inhibitory activities against ACE and PL due to methylation at the B-ring in their molecular structures. Kurita et al. [39] reported that the O-methylated derivative of epigallocatechin gallate exhibited significant inhibitory activity against ACE. The inhibitory activity (IC_50_) exhibited by tangeretin against PL was 148.7 mg/L, which was higher than those exhibited by sinensetin (301.4 mg/L) and nobiletin (426.4 mg/L). Structurally, sinensetin and nobiletin have two methyl groups at the 3′- and 4′- positions of the B-ring, whereas tangeretin has only one methyl group at the 4′-position (Figure 1). Therefore, the methyl group at the 4′-position of the B-ring was assumed to play an important role in the inhibition of PL.

## 4. Conclusions

This study showed that 87.8–98.9% of bioactive flavonoids (hesperidin, narirutin, sinensetin, nobiletin, and tangeretin) could be extracted from *C. unshiu* peel at 145.3–165.6 °C with a water flow rate of 2.25 mL/min in semi-continuous mode using water. Total flavonoid content in the SW extracts was strongly correlated with antioxidant activities (DPPH, FRAP, and ORAC), whereas the sum of HHP in the SW extracts was strongly correlated with inhibitory activities against XO, ACE, α-glucosidase, and PL. The highest antioxidant activities and enzyme inhibitory activities against XO and α-glucosidase were observed for aglycones (hesperetin and naringenin), whereas the highest enzyme inhibitory activities against ACE and PL were observed for monoglucosides of hesperetin and narirutin (i.e., hesperetin-7-O-glucoside and prunin). Therefore, hydrothermal hydrolysate-rich extracts could be used as a functional ingredient in nutraceutical, pharmaceutical, and medicinal industries. Further research on the eco-friendly SW hydrolysis process is required to convert flavonoid diglucosides from citrus peel to lower-molecular-weight compounds with higher functionality.

## Figures and Tables

**Table 1:** Central composite design and corresponding flavonoid and total soluble solid yields in subcritical water extracts from *C. unshiu* peel.

No	X_1_	X_2_	Hesperidin and Its Hydrolysis Products (μg/g Dry Sample)			Narirutin and Its Hydrolysis Products (μg/g Dry Sample)			Polymethoxyflavones (μg/g Dry Sample)			Total Flavonoids (μg/g Dry Sample)	Total Soluble Solids (%)	Total Flavonoid Concentration in the Extract (mg/g Dry Extract)
			Hesperidin	Hesperetin-7-*O*-glucoside	Hesperetin	Narirutin	Prunin	Naringenin	Sinensetin	Nobiletin	Tangeretin			
1	150	1	24,700.4	N.D.	N.D.	5442.7	N.D.	N.D.	13.7	68.8	29.0	30,253.5	45.2 ± 0.3 ^d^	66.6
2	150	2	37,995.1	N.D.	N.D.	8040.6	N.D.	N.D.	18.4	85.4	45.5	46,184.3	56.0 ± 1.7 ^b,c^	82.4
3	170	1	30,498.0	1640.8	1591.0	5631.8	237.8	222.3	13.2	76.4	37.4	39,947.9	58.2 ± 2.4 ^a,b^	68.5
4	170	2	42,975.4	756.8	937.1	8027.2	200.4	145.7	15.7	90.6	49.6	53,197.9	57.9 ± 1.5 ^a,b^	91.8
5	145	1.5	27,935.1	N.D.	N.D.	6687.7	N.D.	N.D.	15.6	76.5	37.5	34,751.6	38.5 ± 1.1 ^e^	90.1
6	175	1.5	34,749.8	2048.0	1802.7	6595.3	326.0	268.1	14.2	82.2	39.1	45,924.3	58.1 ± 0.7 ^a,b^	79.0
7	160	0.75	24,345.7	698.2	433.6	4848.1	162.5	102.3	11.1	62.4	28.2	30,691.3	45.3 ± 1.2 ^d^	67.6
8	160	2.25	44,617.3	370.6	642.5	8760.0	N.D.	135.2	17.7	91.2	54.5	54,688.7	60.6 ± 2.2 ^a^	90.1
9	160	1.5	36,858.1	757.8	870.2	6856.2	141.4	126.1	15.9	82.5	41.6	45,749.5	55.1 ± 0.6 ^c^	82.9
10	160	1.5	36,786.7	701.2	856.7	6832.4	135.9	127.7	16.6	83.9	40.8	45,580.8	---	75.1
11	160	1.5	37,486.7	739.4	884.1	6762.1	142.1	135.1	15.9	83.2	42.1	46,289.9	---	76.3

X_1_: temperature (°C), X_2_: flow rate (mL/min), N.D.: not detected. The same superscript letters in each column (^a–e^) indicate no significant differences (*p* < 0.05).

**Table 2:** Comparison of flavonoids in subcritical water extract and acid and base hydrolysates processed from *C. unshiu* peel.

	Flavonoid Content (μg/g Dry Sample)		
	Subcritical Water Extract *	Acid Hydrolysate	Base Hydrolysate
Hesperidin	34,749.8	12,081.2 ± 1188.3	16,124.1 ± 491.9
Hesperetin-7-*O*-glucoside	2048.0	2462.4 ± 96.0	N.D.
Hesperetin	1802.7	2204.0 ± 39.9	N.D.
Narirutin	6595.3	1240.7 ± 54.2	1620.8 ± 8.3
Prunin	326.0	703.9 ± 23.6	N.D.
Naringenin	268.1	863.0 ± 34.5	N.D.
Sinensetin	14.2	18.3 ± 0.6	15.4 ± 0.3
Nobiletin	82.2	91.1 ± 0.7	74.3 ± 1.7
Tangeretin	39.1	57.6 ± 2.5	41.8 ± 1.1
Total	45,924.3	19,722.2 ± 1236.3	17,876.4 ± 483.9

* Subcritical water extracts were processed at 175 °C and 1.5 mL/min; N.D., not detected. Data are expressed as the mean ± standard deviation of triplicate experiments.

**Table 3:** Antioxidant activities and enzyme inhibitory activities in subcritical water extracts from *C. unshiu* peel.

X_1_	X_2_	Antioxidant Activity			Enzyme Inhibition Activity			
		DPPH Radical Scavenging Activity (mg AAE/g Dry Sample)	FRAP (mmol FSE 100/g Dry Sample))	ORAC (mg TE/g Dry Sample)	Xanthine Oxidase (%)	ACE (%)	α-Glucosidase (%)	Pancreatic Lipase (%)
145	1.5	8.3 ± 0.1 ^f^	24.7 ± 1.1 ^f^	169.5 ± 5.2 ^d^	11.6 ± 1.3 ^f^	12.8 ± 1.5 ^f^	12.8 ± 1.1 ^f^	52.0 ± 1.9 ^g^
150	1	8.2 ± 0.3 ^f^	24.9 ± 0.9 ^f^	122.8 ± 5.8 ^e^	16.6 ± 1.4 ^e^	14.2 ± 0.6 ^e, f^	16.5 ± 0.8 ^e^	59.8 ± 1.9 ^e, f^
150	2	14.8 ± 0.5 ^c^	28.9 ± 0.7 ^e^	338.8 ± 16.6 ^b^	20.8 ± 1.8 ^d^	22.5 ± 1.3 ^d^	21.4 ± 1.4 ^d^	66.8 ± 2.0 ^d^
160	0.75	9.5 ± 0.6 ^e^	25.5 ± 0.9 ^f^	281.3 ± 17.5 ^c^	15.3 ± 1.0 ^e^	16.2 ± 0.7 ^e^	20.7 ± 1.2 ^d^	58.0 ± 1.6 ^f^
160	1.5	13.3 ± 0.7 ^d^	45.3 ± 1.3 ^c^	316.2 ± 26.3 ^b, c^	21.4 ± 0.8 ^d^	21.6 ± 0.5 ^d^	23.1 ± 1.1 ^d^	64.1 ± 2.7 ^d, e^
160	2.25	18.6 ± 0.3 ^a^	48.8 ± 1.3 ^b^	397.4 ± 21.8 ^a^	30.9 ± 1.7 ^c^	35.6 ± 1.5 ^b^	41.0 ± 1.7 ^b, c^	73.3 ± 1.8 ^c^
170	1	14.0 ± 0.0 ^c, d^	39.3 ± 1.1 ^d^	327.6 ± 5.0 ^b^	32.3 ± 1.8 ^c^	32.6 ± 1.7 ^c^	39.2 ± 2.1 ^c^	78.0 ± 1.4 ^b^
170	2	17.2 ± 0.4 ^b^	44.4 ± 1.4 ^c^	423.4 ± 34.9 ^a^	38.1 ± 1.5 ^b^	35.5 ± 1.2 ^b^	42.1 ± 1.2 ^b^	81.3 ± 3.1 ^b^
175	1.5	18.6 ± 0.4 ^a^	52.6 ± 1.8 ^a^	424.9 ± 32.5 ^a^	52.6 ± 2.5 ^a^	48.9 ± 2.6 ^a^	55.5 ± 2.4 ^a^	86.8 ± 3.4 ^a^

X_1_: temperature (°C), X_2_: flow rate (mL/min), DPPH: 2,2-diphenyl-1-picrylhydrazyl, FRAP: ferric-reducing antioxidant power, ORAC: oxygen radical absorbance capacity, ACE: angiotensin-I converting enzyme. The same superscript letters in each column (^a–g^) indicate no significant differences (*p* < 0.05). Data are expressed as the mean ± standard deviation of triplicate experiments.

**Table 4:** Antioxidant activities and enzyme inhibitory activities of individual citrus flavonoids.

	Antioxidant Activity			Enzyme Inhibition Activity (IC_50_)			
	DPPH Radical Scavenging Activity (mg AAE/g)	FRAP (mmol FSE 100/g)	ORAC (mg TE/100 mg)	Xanthine Oxidase (mg/L)	ACE (mg/L)	α-Glucosidase (mg/L)	Pancreatic Lipase (mg/L)
Hesperidin	45.3 ± 0.9 ^c^	399.7 ± 21.1 ^c^	247.3 ± 13.2 ^f^	>2000	1375.8 ± 62.1 ^b^	>2000	418.4 ± 21.7 ^a^
Hesperetin-7-*O*-glucoside	51.6 ± 1.4 ^b^	439.8 ± 18.4 ^b^	323.9 ± 13.7 ^d^	>2000	106.5 ± 3.8 ^f^	1395.5 ± 69.9 ^b^	84.4 ± 4.2 ^d^
Hesperetin	115.8 ± 3.9 ^a^	549.1 ± 21.1 ^a^	710.9 ± 12.0 ^a^	275.4 ± 17.7 ^c^	358.2 ± 7.8 ^d^	131.8 ± 7.3 ^e^	104.6 ± 5.2 ^d^
Narirutin	17.6 ± 1.2 ^e^	102.6 ± 4.1 ^e^	283.4 ± 15.7 ^e^	1104.7 ± 40.7 ^a^	1,937.1 ± 42.5 ^a^	1004.0 ± 42.6 ^c^	>2000
Prunin	26.5 ± 0.2 ^d^	99.5 ± 3.6 ^e^	363.9 ± 18.1 ^c^	862.5 ± 36.6 ^b^	221.2 ± 6.2 ^e^	622.7 ± 28.0 ^d^	>2000
Naringenin	52.6 ± 3.3 ^b^	150.3 ± 3.7 ^d^	475.1 ± 13.3 ^b^	129.3 ± 6.4 ^d^	459.7 ± 15.0 ^c^	72.2 ± 3.8 ^e^	>2000
Sinensetin	N.D.	N.D.	7.6 ± 0.4 ^g^	>2000	364.0 ± 13.4 ^d^	>2000	301.4 ± 22.2 ^b^
Nobiletin	N.D.	N.D.	6.0 ± 0.4 ^g^	>2000	322.5 ± 4.7 ^d^	>2000	426.4 ± 27.6 ^a^
Tangeretin	N.D.	N.D.	5.8 ± 0.4 ^g^	>2000	352.0 ± 8.9 ^d^	1763.1 ± 66.6 ^a^	148.7 ± 6.5 ^c^
Positive control				116.7 ± 3.7 ^d^ (Allopurinol)	6.9 ± 0.2 ^g^ (Captopril)	1366.9 ± 73.2 ^b^ (Acarbose)	54.4 ± 3.6 ^e^ (Orlistat)

DPPH: 2,2-diphenyl-1-picrylhydrazyl, FRAP: ferric-reducing antioxidant power, ORAC: oxygen radical absorbance capacity, ACE: angiotensin-I converting enzyme. The same superscript letters in each column (^a–g^) indicate no significant differences (*p* < 0.05). Data are expressed as the mean ± standard deviation of triplicate experiments.

## Supplementary Materials

The following are available online at https://www.mdpi.com/2076-3921/9/5/360/s1, Figure S1: Schematic diagram of the dynamic subcritical water extraction apparatus (CV, check valve; EV, extraction vessel; F, in-line filter; FM, flow meter; HE, heat exchanger; HPP, high pressure pump; MV, metering valve; P, pressure gauge; PC, preheating coil; PR, pressure regulator; SV, safety valve; SR, solvent reservoir; T, temperature indicator; V, on/off valve), Figure S2: High-performance liquid chromatography chromatograms of the (a) methanol extract, (b) acid hydrolysate, and (c) base hydrolysate of *Citrus unshiu* peel, Figure S3: High-performance liquid chromatography chromatograms of flavonoid standards, Figure S4: High-performance liquid chromatography chromatograms of the subcritical water extract at 175 °C and 1.5 mL/min, Table S1: The regression equation for each standard compound analyzed, Table S2: Symbols and levels of the independent variables, Table S3: Composition of flavonoids in *Citrus unshiu* peel, Table S4: Results of analysis of variance for regression models, Table S5: Optimal subcritical water extraction parameters for maximizing flavonoid yields from *C. unshiu* peel, Table S6: Pearson correlation coefficients representing associations between flavonoid yields and biological activities, Table S7: Theoretical and measured antioxidant activities in subcritical water extracts, Table S8: Xanthine oxidase inhibitory activities at different flavonoid concentrations, Table S9: Angiotensin-I converting enzyme inhibitory activities at different flavonoid concentrations, Table S10: α-Glucosidase inhibitory activities at different flavonoid concentrations, Table S11: Pancreatic lipase inhibitory activities at different flavonoid concentrations.
