# Supplementary material for: Semi-Continuous Subcritical Water Extraction of Flavonoids from Citrus unshiu Peel: Their Antioxidant and Enzyme Inhibitory Activities
Source: Antioxidants (Basel). 2020 Apr 25;9(5):360. doi: 10.3390/antiox9050360 (PMC7278842; doi:10.3390/antiox9050360)
Supplement: Supplementary file 1 [file antioxidants-09-00360-s001.pdf]

Supplementary materials

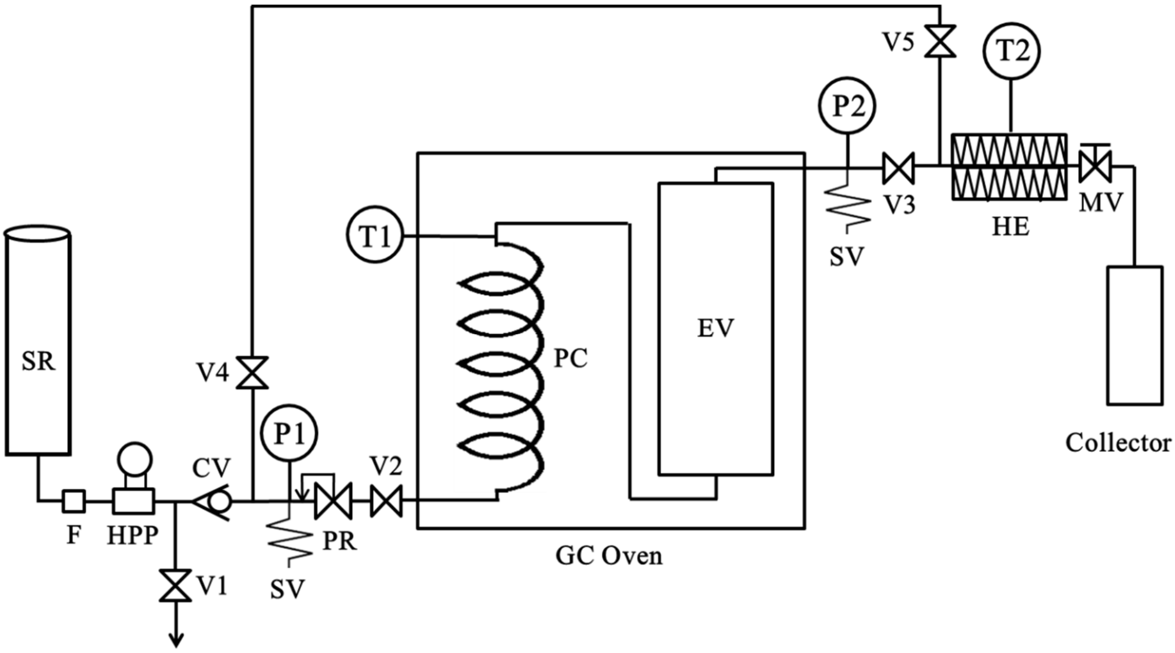

Figure S1. Schematic diagram of the dynamic subcritical water extraction apparatus (CV, check valve; EV, extraction vessel; F, in-line filter; FM, flow meter; HE, heat exchanger; HPP, high pressure pump; MV, metering valve; P, pressure gauge; PC, preheating coil; PR, pressure regulator; SV, safety valve; SR, solvent reservoir; T, temperature indicator; V, on/off valve).

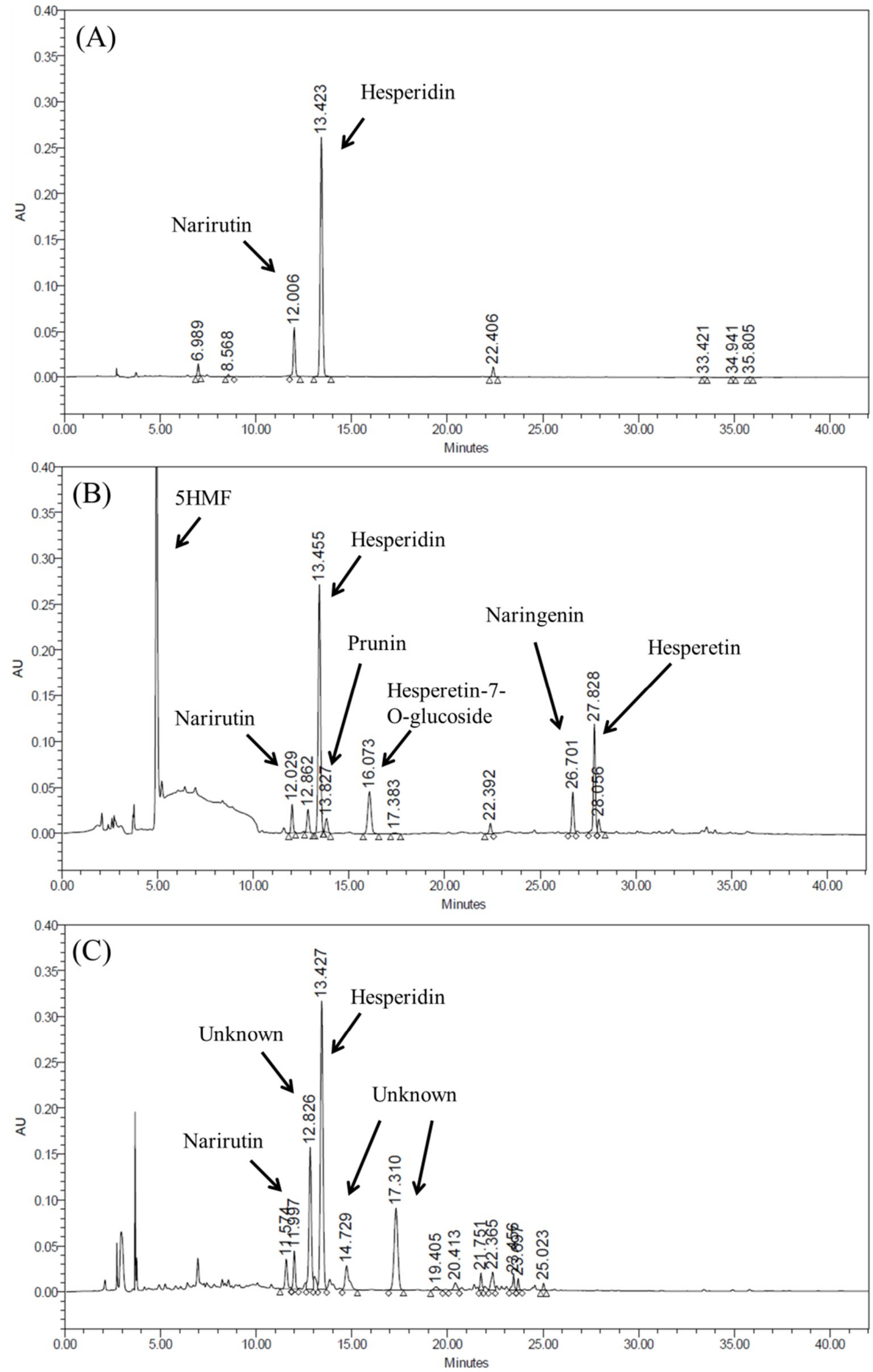

Figure S2. High-performance liquid chromatography chromatograms of the (a) methanol extract, (b) acid hydrolysate, and (c) base hydrolysate of *Citrus unshiu* peel.

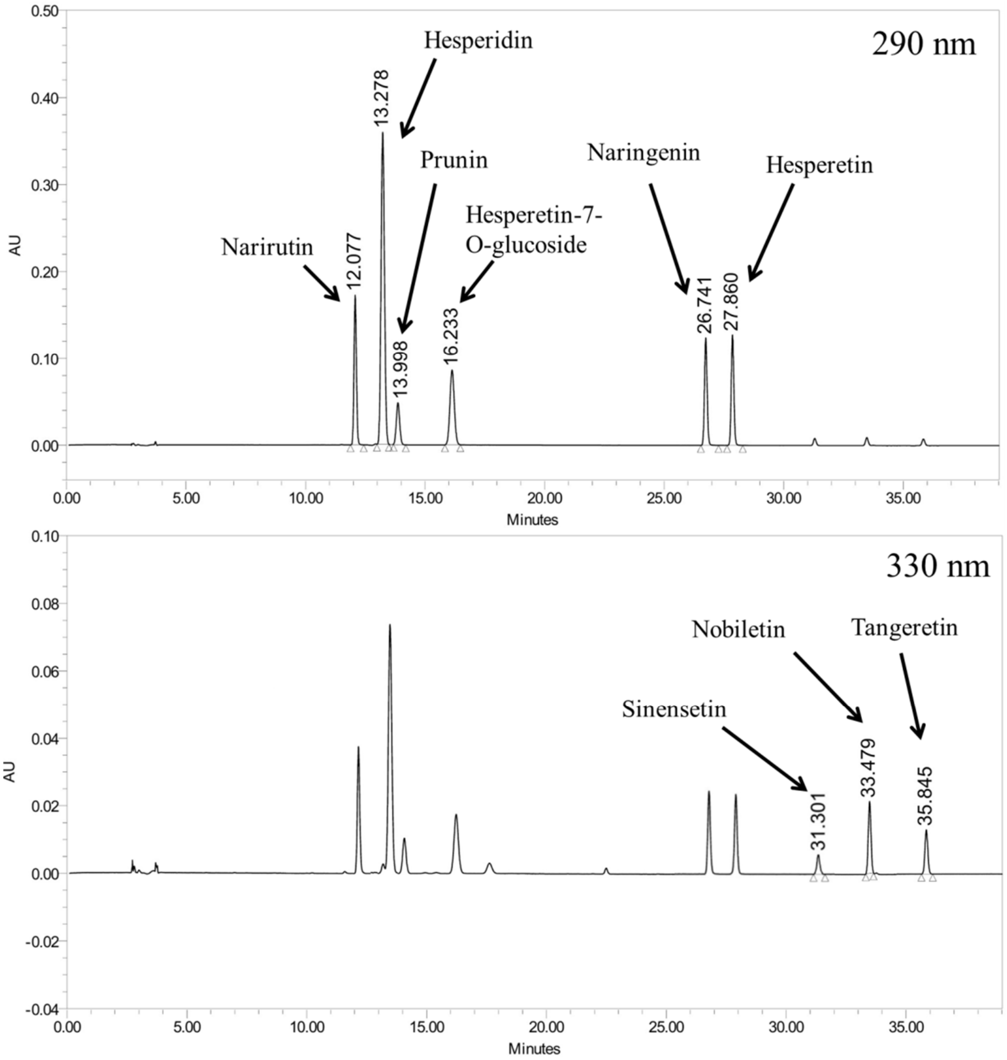

Figure S3. High-performance liquid chromatography chromatograms of flavonoid standards.

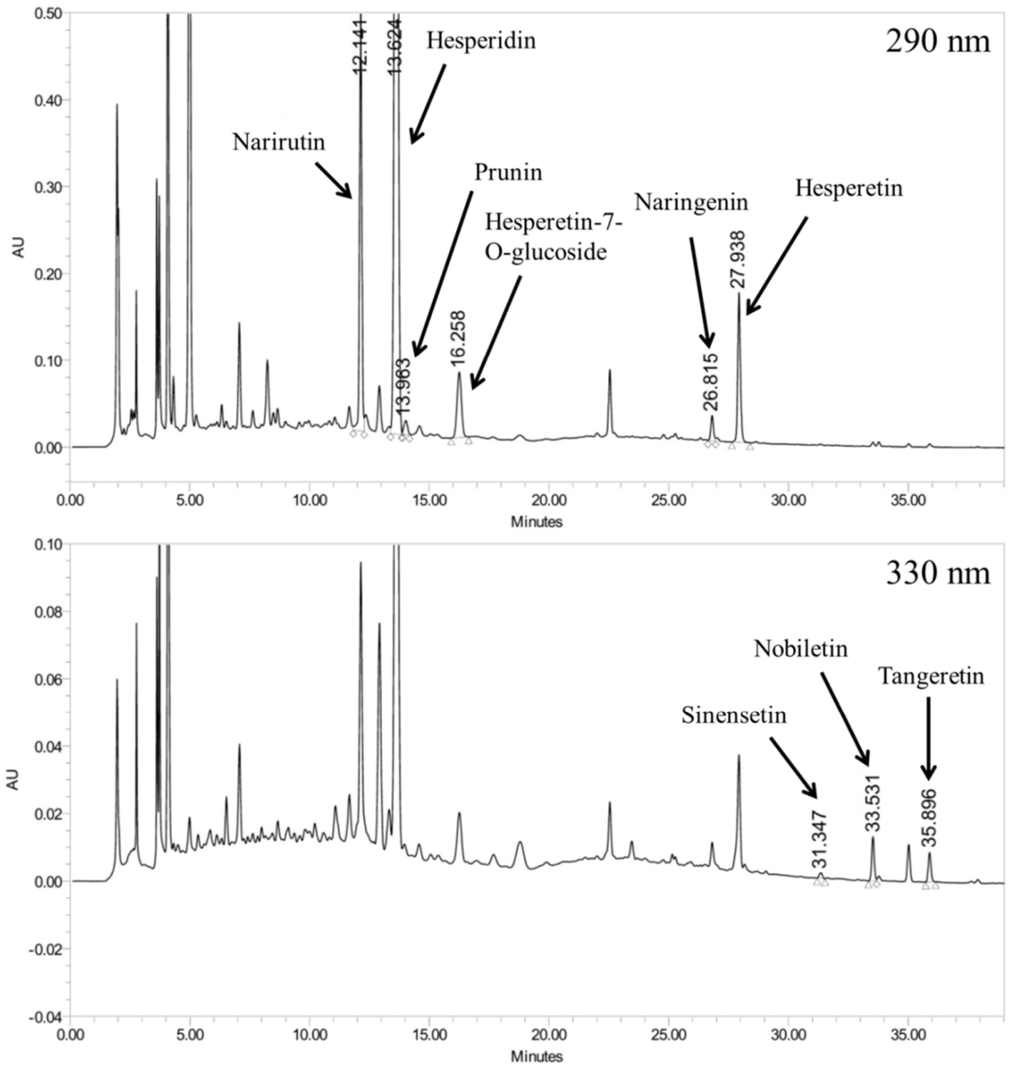

Figure S4. High-performance liquid chromatography chromatograms of the subcritical water extract at 175 °C and 1.5 mL/min.

Table S1. The regression equation for each standard compound analyzed.

Compound	Linear range (mg/L)	Calibration curve	R^2
Hesperidin	25–200	$y = 18.063x + 6.25$	0.9996
Hesperetin-7-*O*-glucoside	6–50	$y = 21.997x - 0.1071$	0.9999
Hesperetin	5–25	$y = 38.8x - 3$	0.9999
Narirutin	10–80	$y = 16.423x - 0.1429$	0.9994
Prunin	2.5–20	$y = 23.938x + 0.0714$	0.9998
Naringenin	5–25	$y = 38.34x - 2.1$	0.9999
Sinensetin	0.2–1	$y = 42.5x - 0.7$	0.9996
Nobiletin	0.5–5	$y = 41.762x + 0.0357$	0.9998
Tangeretin	0.25–2.25	$y = 54.381x + 0.0714$	0.9997

Table S2. Symbols and levels of the independent variables.

	Symbol	Factor level				
		**−1.5**	**−1**	**0**	**+1**	**+1.5**
Temperature (°C)	X_1	145	150	160	170	175
Flow rate (mL/min)	X_2	0.75	1	1.5	2	2.25

Table S3. Composition of flavonoids in *Citrus unshiu* peel.

	Flavonoid content	
	**(μg/g dry sample)**	**(%)**
Hesperidin	50,027.4 ± 970.8	84.1
Narirutin	9284.4 ± 241.1	15.6
Sinensetin	18.9 ± 0.2	0.03
Nobiletin	103.8 ± 0.5	0.18
Tangeretin	55.5 ± 0.3	0.09
Total	59,490.0 ± 1211.5	100

Data are expressed as the mean ± standard deviation of triplicate experiments.

Table S4. Results of analysis of variance for regression models.

Source	Hesperidin		Narirutin		Sinensetin		Nobiletin		Tangeretin	
	F-value	*p*-value (Prob > F)	*F*-value	*p*-value (Prob > F)	*F*-value	*p*-value (Prob > F)	*F*-value	*p*-value (Prob > F)	*F*-value	*p*-value (Prob > F)
Model	245.79	< 0.0001	476.95	< 0.0001	33.03	0.0008	41.40	0.0005	28.62	0.0011
X_1	137.85	< 0.0001	0.03	0.8708	12.67	0.0162	14.87	0.0119	6.36	0.0531
X_2	986.57	< 0.0001	2376.63	< 0.0001	133.22	< 0.0001	178.96	< 0.0001	132.15	< 0.0001
X_1^2	103.29	0.0002	5.37	0.0682	6.31	0.0537	3.87	0.1064	2.98	0.1447
X_2^2	18.35	0.0078	0.02	0.9010	12.59	0.0164	12.05	0.0178	0.00	0.9814
X_1X_2	0.44	0.5348	1.76	0.2425	4.74	0.0814	0.39	0.5600	1.17	0.3284
Lack of fit	3.56	0.2270	3.40	0.2355	1.92	0.3602	11.20	0.0831	13.71	0.0688
R^2	0.9959		0.9979		0.9706		0.9764		0.9662	
pred R^2	0.9742		0.9867		0.8267		0.8371		0.7641	
adj R^2	0.9919		0.9958		0.9412		0.9528		0.9325	
C.V%	1.78		1.13		3.33		2.36		5.02	
Adeq precision	44.8		67.9		17.5		18.64		16.1	

X_1: temperature (°C), X_2: flow rate (mL/min), pred R^2: predicted R^2, adj R^2: adjusted R^2, C.V: coefficient of variance, Adeq precision: adequate precision.

Table S5. Optimal subcritical water extraction parameters for maximizing flavonoid yields from *C. unshiu* peel.

	Temperature (°C)	Flow rate (mL/min)	Predicted yield		Desirability
			μg/g dry sample	%	
Hesperidin (H)	164.4	2.25	45,211.0	90.4	1
Narirutin (N)	154.6	2.25	8765.2	94.4	1
Sinensetin (S)	145.3	2.25	18.7	98.9	1
Nobiletin (NO)	165.6	2.25	91.2	87.8	1
Tangeretin (T)	160.5	2.25	53.6	96.6	1
H+N	159.5	2.25	44,622.4/8,749.7	89.1/ 94.2	1
S+NO+T	153.5	2.25	18.4/89.0/52.9	97.3/85.7 /95.3	0.8603
H+N+S+NO+T	158.5	2.25	44,366.4/8,755.4 /17.8/90.4/53.5	88.7/94.3 /94.2/87.1 /96.4	0.6389

Table S6. Pearson correlation coefficients representing associations between flavonoid yields and biological activities.

	Antioxidant activity			Enzyme inhibition activity			
	**DPPH radical scavenging activity**	**FRAP**	**ORAC**	**Xanthine oxidase**	**ACE**	**α-Glucosidase**	**Pancreatic lipase**
Sum of all flavonoids	0.911**	0.798**	0.841**	0.615*	0.698*	0.647	0.676*
Sum of hydrolysis products	0.567	0.719*	0.629	0.826**	0.798**	0.830**	0.795*
Hesperidin (HPD)	0.857**	0.725*	0.782*	0.513	0.602	0.549	0.585
H7G (HPG)	0.505	0.651	0.576	0.796*	0.761*	0.793*	0.753*
Hesperetin (HPT)	0.629	0.786*	0.666	0.848**	0.831**	0.860**	0.830**
HPD+HPG+HPT	0.932**	0.822**	0.859**	0.639	0.719*	0.673*	0.702*
HPG+HPT	0.569	0.721	0.624	0.827**	0.801**	0.831**	0.796*
Narirutin (NRT)	0.685*	0.479	0.560	0.293	0.393	0.314	0.344
Prunin (PRN)	0.419	0.562	0.562	0.736*	0.666*	0.708*	0.695*
Naringenin (NGN)	0.664	0.812**	0.718*	0.845**	0.847**	0.887**	0.821**
NRT+PRN+NGN	0.786*	0.601	0.676*	0.432	0.526	0.453	0.477
PRN+NGN	0.543	0.692*	0.650	0.808**	0.768*	0.809**	0.773*
Sinensetin	0.459	0.269	0.292	0.047	0.139	0.029	0.102
Nobiletin	0.812**	0.696*	0.689*	0.518	0.591	0.520	0.571
Tangeretin	0.787*	0.625	0.690*	0.411	0.522	0.466	0.485
Total PMFs	0.782*	0.634	0.662	0.431	0.525	0.454	0.495

* $p < 0.05$ and ** $p < 0.01$, sum of hydrolysis products: HPG+HPT+ PRN+NGN, DPPH: 2,2-diphenyl-1-picrylhydrazyl, FRAP: ferric-reducing antioxidant power, ORAC: oxygen radical absorbance capacity, ACE: angiotensin-I converting enzyme, PMFs: polymethoxyflavones.

Table S7. Theoretical and measured antioxidant activities in subcritical water extracts.

X_1	X_2	DPPH radical scavenging activity (mg AAE/g)		FRAP (mmol FSE/100 g)		ORAC (mg TE/g)	
		**Theoretical**	**Measured**	**Theoretical**	**Measured**	**Theoretical**	**Measured**
145	1.5	1.4	8.3 ± 0.1^{f}	11.9	24.7 ± 1.1^{f}	88.0	169.5 ± 5.2^{d}
150	1	1.2	8.2 ± 0.3^{f}	10.4	24.9 ± 0.9^{f}	76.5	122.8 ± 5.8^{e}
150	2	1.9	14.8 ± 0.5^{c}	16.0	28.9 ± 0.7^{e}	116.8	338.8 ± 16.6^{b}
160	0.75	1.3	9.5 ± 0.6^{e}	10.8	25.5 ± 0.9^{f}	80.4	281.3 ± 17.5^{c}
160	1.5	1.9	13.3 ± 0.7^{d}	16.3	45.3 ± 1.3^{c}	120.3	316.2 ± 26.3^{bc}
160	2.25	2.3	18.6 ± 0.3^{a}	19.3	48.8 ± 1.3^{b}	141.6	397.4 ± 21.8^{a}
170	1	1.8	14.0 ± 0.0^{cd}	14.4	39.3 ± 1.1^{d}	109.9	327.6 ± 5.0^{b}
170	2	2.2	17.2 ± 0.4^{b}	18.9	44.4 ± 1.4^{c}	139.6	423.4 ± 34.9^{a}
175	1.5	2.0	18.6 ± 0.4^{a}	16.5	52.6 ± 1.8^{a}	126.5	424.9 ± 32.5^{a}

X_1: temperature (°C), X_2: flow rate (mL/min), DPPH: 2,2-diphenyl-1-picrylhydrazyl, FRAP: ferric-reducing antioxidant power, ORAC: oxygen radical absorbance capacity. The same superscript letters in each column ($^{a–f}$) indicate no significant differences ($p < 0.05$). Data are expressed as the mean ± standard deviation of triplicate experiments.

Table S8. Xanthine oxidase inhibitory activities at different flavonoid concentrations.

Flavonoid	Concentration (mg/L)	Inhibitory activity (%)
Hesperetin	150	40.1 ± 1.2
	200	44.1 ± 1.2
	250	18.1 ± 1.2
	300	51.6 ± 2.6
	350	56.1 ± 1.4
Narirutin	600	42.3 ± 1.3
	800	45.4 ± 0.8
	1000	48.2 ± 0.9
	1200	51.3 ± 0.5
	1400	54.4 ± 1.5
Prunin	400	42.9 ± 0.5
	600	46.1 ± 0.7
	800	48.8 ± 0.1
	1000	52.6 ± 0.8
	1200	55.0 ± 0.9
Naringenin	120	48.3 ± 1.1
	140	52.3 ± 0.8
	160	55.2 ± 1.8
	180	59.1 ± 2.9
	200	62.6 ± 2.0
Allopurinol (positive control)	80	33.8 ± 1.6
	100	42.7 ± 0.3
	120	51.6 ± 2.2
	140	61.7 ± 4.1
	160	68.1 ± 2.8

Data are expressed as the mean ± standard deviation of triplicate experiments.

Table S9. Angiotensin-I converting enzyme inhibitory activities at different flavonoid concentrations.

Flavonoid	Concentration (mg/L)	Inhibitory activity (%)
Hesperidin	1000	40.1 ± 1.2
	1200	46.2 ± 1.3
	1400	51.2 ± 1.7
	1600	55.6 ± 1.6
	1800	60.2 ± 3.4
Hesperetin-7-*O*-glucoside	60	37.4 ± 1.9
	80	43.6 ± 0.7
	100	48.3 ± 1.0
	120	53.3 ± 1.5
	140	58.7 ± 0.3
Hesperetin	100	27.9 ± 0.2
	200	36.3 ± 0.2
	300	46.4 ± 2.0
	400	54.4 ± 0.3
	500	60.7 ± 0.9
Narirutin	1200	32.8 ± 0.9
	1400	36.6 ± 0.5
	1600	41.8 ± 0.9
	1800	47.2 ± 1.2
	2000	51.4 ± 1.1
Prunin	100	35.7 ± 0.5
	150	42.2 ± 0.9
	200	47.4 ± 1.0
	250	52.8 ± 0.6
	300	59.7 ± 1.3
Naringenin	200	33.2 ± 1.1
	300	39.5 ± 0.9
	400	46.4 ± 1.1
	500	52.8 ± 1.0
	600	59.0 ± 1.8
Sinensetin	100	12.2 ± 1.4
	200	28.5 ± 0.8
	300	41.4 ± 1.3
	400	55.5 ± 0.9
	500	65.8 ± 2.2
Nobiletin	100	24.4 ± 0.8
	200	35.9 ± 0.3
	300	45.5 ± 1.4
	400	59.7 ± 1.1
	500	71.3 ± 1.7
Tangeretin	100	23.5 ± 1.1
	200	33.8 ± 1.4
	300	45.9 ± 1.6
	400	53.6 ± 0.5
	500	66.1 ± 1.9
Captoril (positive control)	2	8.3 ± 2.7
	4	12.3 ± 2.1
	6	40.2 ± 2.7
	8	63.4 ± 2.5
	10	89.0 ± 2.8

Data are expressed as the mean ± standard deviation of triplicate experiments.

Table S10. *α*-Glucosidase inhibitory activities at different flavonoid concentrations.

Flavonoid	Concentration (mg/L)	Inhibitory activity (%)
Hesperetin-7-*O*-glucoside	1000	41.1 ± 1.3
	1200	45.0 ± 1.5
	1400	50.5 ± 2.4
	1600	54.7 ± 1.5
	1800	59.2 ± 1.8
Hesperetin	50	39.0 ± 2.0
	75	42.7 ± 2.1
	100	45.9 ± 1.5
	125	49.0 ± 1.1
	150	52.2 ± 0.7
Narirutin	400	33.9 ± 2.5
	600	39.5 ± 1.3
	800	44.0 ± 0.4
	1000	49.8 ± 1.7
	1200	55.5 ± 1.1
Prunin	400	41.6 ± 1.1
	500	45.6 ± 1.3
	600	49.5 ± 2.0
	700	52.4 ± 0.9
	800	56.7 ± 0.5
Naringenin	50	46.3 ± 0.8
	75	50.4 ± 0.7
	100	54.9 ± 1.1
	125	58.9 ± 1.4
	150	63.6 ± 2.3
Tangeretin	1200	41.3 ± 1.1
	1400	44.7 ± 1.1
	1600	47.2 ± 0.9
	1800	50.7 ± 0.9
	2000	53.4 ± 1.8
Acarbose (positive control)	1000	44.5 ± 1.7
	1200	47.9 ± 1.2
	1400	50.2 ± 0.8
	1600	53.2 ± 1.1
	1800	56.6 ± 1.2

Data are expressed as the mean ± standard deviation of triplicate experiments.

Table S11. Pancreatic lipase inhibitory activities at different flavonoid concentrations.

Flavonoid	Concentration (mg/L)	Inhibitory activity (%)
Hesperidin	200	43.7 ± 1.6
	400	50.0 ± 0.8
	600	55.0 ± 0.4
	800	60.1 ± 0.4
	1,000	64.8 ± 1.6
Hesperetin-7-*O*-glucoside	50	46.7 ± 0.3
	100	51.4 ± 0.5
	150	56.2 ± 1.8
	200	61.5 ± 1.0
	250	65.7 ± 1.8
Hesperetin	50	45.7 ± 1.0
	100	49.9 ± 0.4
	150	53.3 ± 0.6
	200	57.3 ± 0.3
	250	60.4 ± 0.9
Sinensetin	100	40.3 ± 2.9
	200	46.0 ± 1.9
	300	50.3 ± 0.9
	400	54.4 ± 0.9
	500	58.2 ± 1.9
Nobiletin	100	35.1 ± 1.6
	200	39.4 ± 0.9
	300	44.6 ± 1.5
	400	48.1 ± 1.1
	500	53.8 ± 1.4
Tangeretin	50	37.9 ± 0.8
	100	43.8 ± 0.5
	150	51.0 ± 0.7
	200	55.9 ± 0.8
	250	62.1 ± 1.8
Orlistat (positive control)	20	40.1 ± 4.1
	40	44.6 ± 2.5
	60	51.7 ± 1.2
	80	57.8 ± 1.6
	100	63.0 ± 2.7

Data are expressed as the mean ± standard deviation of triplicate experiments.
